# Genetic resistance determinants to fusidic acid and chlorhexidine in variably susceptible staphylococci from dogs

**DOI:** 10.1186/s12866-019-1449-z

**Published:** 2019-04-25

**Authors:** S-M Frosini, R. Bond, M. Rantala, T. Grönthal, S. C. Rankin, K. O’Shea, D. Timofte, V. Schmidt, J. Lindsay, A. Loeffler

**Affiliations:** 1 Department of Clinical Sciences and Services, Royal Veterinary College, Hawkshead Lane, North Mymms, Hatfield, Hertfordshire, AL9 7TA UK; 2 Department of Equine and Small Animal Medicine, Faculty of Veterinary Medicine, University of Helsinki, P.O. Box 57, 00014 Helsinki, Finland; 3 Department of Pathobiology, School of Veterinary Medicine, University of Pennsylvania, 3850 Spruce Street, Philadelphia, PA 19104 USA; 4 Institute of Veterinary Science, University of Liverpool, Chester High Road, Neston, CH64 7TE UK; 5 Institute of Infection and Immunity, St George’s, University of London, Cranmer Terrace, London, SW17 0RE UK

**Keywords:** Staphylococci, Canine, Fusidic acid, Chlorhexidine, Resistance, Veterinary

## Abstract

**Background:**

Concern exists that frequent use of topically-applied fusidic acid (FA) and chlorhexidine (CHX) for canine pyoderma is driving clinically relevant resistance, despite rare description of FA and CHX genetic resistance determinants in canine-derived staphylococci. This study aimed to determine minimum inhibitory concentrations (MICs) and investigate presence of putative resistance determinants for FA and CHX in canine-derived methicillin-resistant (MR) and -susceptible (MS) staphylococci. Plasmid-mediated resistance genes (*fusB, fusC, fusD, qacA/B, smr;* PCR) and MICs (agar dilution) of FA and CHX were investigated in 578 staphylococci (50 MR *S. aureus* [SA], 50 MSSA, 259 MR *S. pseudintermedius* [SP], 219 MSSP) from Finland, U.S.A., North (NUK) and South-East U.K. (SEUK) and Germany. In all isolates with FA MIC ≥64 mg/L (*n* = 27) *fusA* and *fusE* were amplified and sequenced.

**Results:**

FA resistance determinants (*fusA* mutations *n* = 24, *fusB* n = 2, *fusC n* = 36) were found in isolates from all countries bar U.S.A. and correlated with higher MICs (≥1 mg/L), although 4 SP isolates had MICs of 0.06 mg/L despite carrying *fusC*. CHX MICs did not correlate with *qacA/B* (n = 2) and *smr* (*n* = 5), which were found in SEUK SA, and SP from NUK and U.S.A.

**Conclusions:**

Increased FA MICs were frequently associated with *fusA* mutations and *fusC,* and this is the first account of *fusB* in SP. Despite novel description of *qacA/B* in SP, gene presence did not correlate with CHX MIC. Selection pressure from clinical use might increase prevalence of these genetic determinants, but clinical significance remains uncertain in relation to high skin concentrations achieved by topical therapy.

## Background

Coagulase-positive staphylococci, primarily *Staphylococcus pseudintermedius* and less often *S. aureus* and *S. schleiferi,* are the predominant pathogens in canine superficial pyoderma [1]. The emergence of methicillin-resistant strains of *S. pseudintermedius* (MRSP), that are usually resistant to most or all available licensed systemic veterinary antimicrobials [2, 3], has increased interest in the use of topical therapy [4], most commonly with products that contain fusidic acid or chlorhexidine [5]. These same antimicrobials are used topically in human medicine, but in people fusidic acid is also used systemically as a last line treatment option for bacteraemia caused by methicillin-resistant *S. aureus* (MRSA) [6]*.*

Infections caused by methicillin-susceptible (MS) and MRSP have been documented in humans [7–10], and zoonotic transmission of MSSP and MRSP has been inferred by the isolation of genetically identical MRSP isolates from pet dogs and their infected human owners [11]. Similarly, occasional human nasal carriage of MSSP and even MRSP has been described [12–14], again with indistinguishable pulsed-field gel electrophoresis (PFGE) patterns to those carried by in-contact pet dogs [15, 16].

Topical therapy with fusidic acid is common in human medicine for staphylococcal skin infections and is also recommended [17] and used in dogs with skin infections, at least in European countries but not in the U.S.A. Chlorhexidine is used worldwide as a disinfectant and antiseptic, and in topical antibacterial products for dogs. Whilst topical antibacterial therapy is recommended as an alternative to systemic treatment [4] in order to reduce selection pressure on pathogens, there are concerns over reduced phenotypic susceptibility to these agents [18, 19]. In New Zealand, clonal expansion of fusidic acid-resistant *S. aureus* (based on disk diffusion testing) was reported concurrently with a significant increase in national dispensing of topical fusidic acid products for humans [20]. By contrast, the prevalence of phenotypic resistance to fusidic acid (minimum inhibitory concentration [MIC] ≥ 1 mg/L determined using VITEK 2) [21] increased amongst MRSA in the U.K. despite stable (2002–2009) and decreasing (2009–2013) fusidic acid sales [22]. Reduced susceptibility of MRSA to chlorhexidine following increased antiseptic use was demonstrated in human hospitals [19, 23, 24]; the presence of genetic characteristics thought to be related to reduced chlorhexidine susceptibility has also been implicated in failure of decolonisation strategies [25].

The acquired resistance genes *fusB* [26–28], *fusC* [27–29] and *fusD* [28, 29]*,* most commonly carried on plasmids, and chromosomal mutations in *fusA* [27, 28, 30, 31] and *fusE* [28, 31] have been associated with reduced susceptibility to fusidic acid in *S. aureus.* Geographical variation in the presence of these genes in phenotypically fusidic acid-resistant *S. aureus* derived from humans, defined by clinical breakpoints, has been described [28, 32–34]. In *S. pseudintermedius*, there is one publication that describes *fusA* mutations conferring fusidic acid resistance in a single isolate [35], and only two isolates of *S. (pseud)intermedius* have been shown to carry *fusC* [29], despite widespread licensing and marketing of fusidic acid products for topical use in small animal veterinary practice in Europe during the past four decades.

Plasmid-derived *qacA/B* and *smr* have an uncertain correlation with reduced susceptibility to chlorhexidine amongst staphylococci [36–41]. Transfer of *qacA/B* by transduction between isolates of *S. aureus* has been described, although the effect of this transfer on susceptibility to chlorhexidine was not assessed [42]*.* Whether transfer of resistance genes can occur between *S. pseudintermedius* and *S. aureus* still remains unclear but evidence for such transfer between staphylococcal species exists [43], most notably of the SCC*mec* (predominantly type IV) which encodes methicillin resistance and is believed to have originated in coagulase-negative staphylococci [44–47]. An increase in the prevalence of resistance genes in canine-derived staphylococci due to veterinary use of topically-applied antimicrobials could become of concern to veterinarians if clinical failure occurred. Furthermore, there could be implications for both human and canine health through either transfer of resistant strains between hosts, or of genetic material to susceptible bacterial species. This study investigated the association between resistance genes and MICs of fusidic acid and chlorhexidine in canine-derived *S. pseudintermedius* and *S. aureus* in a large collection of isolates obtained from wide geographical areas.

## Results

**Fig. 1:**
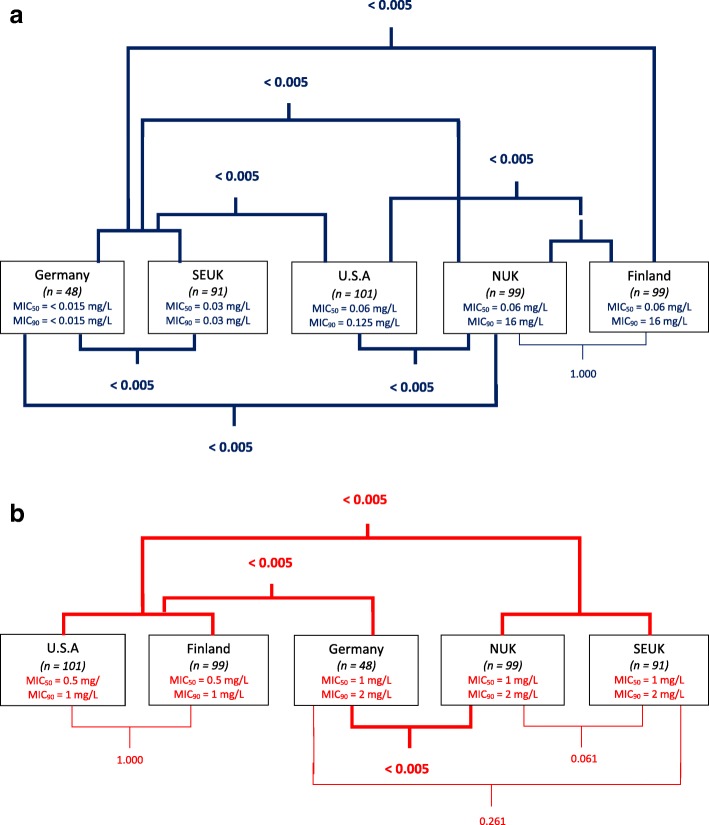
Comparative statistical overview of MIC of **a**) fusidic acid and **b**) chlorhexidine for canine-derived *S. pseudintermedius* from different geographical regions. *P* values stated; *P* < 0.05 indicates significance, depicted in bold. SEUK: South-East U.K.; NUK: North U.K

The MICs (new and previously determined), MIC_50,_ MIC_90_ values and comparisons between regional groups are detailed in Fig. 1 and Tables 1 and 2.

**Table 1:** Minimum inhibitory concentrations (MICs) of fusidic acid determined by agar dilution, and presence of resistance determinants, for canine-derived *Staphylococcus pseudintermedius* and *S. aureus* isolates (*n* = 578) from Finland, the U.S.A., North U.K. (NUK), South-East U.K. (SEUK) and Germany

Country	Bacterial Type	n	Fusidic acid MIC (mg/L)														MIC_50_	MIC_90_
			≤0.015	0.03	0.06	0.125	0.25	0.5	1	2	4	8	16	32	64	>64	(mg/L)	(mg/L)
Finland	MRSP	49	0	0	28 n = 2 *fusC*	0	0	0	0	3	5 n = 4 *fusC*	4 n = 2 *fusC*	8 n = 1 *fusC*	0	1 n = 1 *fusA*	0	0.06	16
	FA-R MRSP	40	0	0	0	0	0	0	0	0	13 n = 12 *fusC*	10 n = 1 *fusC* n = 2 *fusB*	5	0	12 n = 12 *fusA*	0	8	64
	MSSP	50	0	1	38 n = 1 *fusC*	1	0	0	0	0	0	7 n = 3 *fusC*	3 n = 1 *fusC*	0	0	0	0.06	8
U.S.A.	MRSP	50	0	2	38	10	0	0	0	0	0	0	0	0	0	0	0.06	0.125
	MSSP	51	0	0	47	2	0	0	0	0	2	0	0	0	0	0	0.06	0.06
NUK	MRSP	49	0	0	14 n = 1 *fusC*	4	0	0	0	14	2 n = 1 *fusC*	10 n = 5 *fusC*	0	0	4 n = 4 *fusA*	1	2	64
	MSSP	50	0	1	37	0	0	0	1	0	1	5	1	0	2 n = 2 *fusA*	2 n = 2 *fusA*	0.06	8
SEUK^a^	MRSP	47	22	1	12	3	0	0	1	2	4	0	1	0	1 n = 1 *fusA*	0	0.06	4
	MSSP	44	19	5	14	1	0	0	2	2	0	1	0	0	0	0	0.03	1
	MRSA	50	8	34	0	0	3	0	0	0	0	0	0	0	1	4 n = 2 *fusA*	0.03	0.25
	MSSA	50	24	13	0	0	1	1	6	2	2 n = 2 *fusC*	0	1	0	0	0	0.03	1
Germany^a^	MRSP	24	0	0	0	0	0	0	0	0	0	0	0	0	0	0	<0.015	<0.015
	MSSP	21	0	0	0	0	0	0	0	3	0	0	0	0	0	0	<0.015	<0.015

EUCAST breakpoint for fusidic acid for staphylococci is 1 mg/L; (reference [62]).

*MRSP* methicillin-resistant *Staphylococcus pseudintermedius*, *FA-R* fusidic acid-resistant, *MSSP* methicillin-sensitive *S. pseudintermedius*, *NUK* North U.K., *SEUK* South-East U.K, *MRSA* methicillin-resistant *S. aureus*, *MSSA* methicillin-susceptible *S. aureus.*

^a^MICs determined as part of previous study by the authors (reference [52])

**Table 2:** Minimum inhibitory concentrations (MICs) of chlorhexidine determined by agar dilution, and presence of resistance determinants, for canine-derived *Staphylococcus pseudintermedius* and *S. aureus* isolates (*n* = 538) from Finland, the U.S.A., North U.K. (NUK), South-East U.K. (SEUK) and Germany

Country	Bacterial Type	n	Chlorhexidine MIC (mg/L)								MIC_50_	MIC_90_
			0.125	0.25	0.5	1	2	4	8	16	mg/L	mg/L
Finland	MRSP	49	0	1	35	12	0	1	0	0	0.5	1
	MSSP	50	0	0	38	12	0	0	0	0	0.5	1
U.S.A.	MRSP	50	0	0	35 n = 2 *smr*	15 n = 1 *smr*	0	0	0	0	0.5	1
	MSSP	51	0	0	45 n = 1 *smr*	4	1	1	0	0	0.5	1
NUK^b^	MRSP	49	0	0	0	16	25	8	0	0	2	4
	MSSP	50	0	0	0	36	14 n = 1 *qacA/B*	0	0	0	1	2
SEUK^a^	MRSP	47	0	0	0	21	23	2	1	0	2	2
	MSSP	44	0	0	1	22	17	2	2	0	1	2
	MRSA	50	0	0	0	0	0	49	1	0	4	4
	MSSA	50	0	0	1	22	22	5 n = 1 *qacA/B* n = 1 *smr*	0	0	2	2
Germany^a^	MRSP	24	0	0	9	4	11	0	0	0	1	2
	MSSP	24	0	0	11	13	0	0	0	0	1	1

*MRSP* methicillin-resistant *Staphylococcus pseudintermedius*, *MSSP* methicillin-sensitive *S. pseudintermedius*, *NUK* North U.K., *SEUK* South-East U.K, *MRSA* methicillin-resistant *S. aureus*, *MSSA* methicillin-susceptible *S. aureus.*

^a^MICs determined as part of previous study by the authors (reference [52])

^b^MICs determined as part of previous study by the authors (reference [45])

The MICs of fusidic acid specifically determined for this study for *S. pseudintermedius* (*n* = 339) from NUK, Finland and the U.S.A ranged from 0.03 to > 64 mg/L. In the 40 Finnish FA-R MRSP the lowest MIC was 4 mg/L. Of the remaining 299 isolates, 76 had MIC ≥1 mg/L (43 NUK, 31 Finland, 2 U.S.A) while 223 isolates were phenotypically fusidic acid-susceptible based on EUCAST breakpoints [21] (Table 1). The MICs of reference isolates were low (ATCC® 25923™ and LMG 22219, 0.06 mg/L; ATCC® 29663™, 0.03 mg/L), consistent with previous reports [48, 49]. Chlorhexidine MICs of Finnish and U.S.A. isolates (*n* = 200) ranged from 0.25 to 4 mg/L; 196 isolates had MICs of 0.5 or 1 mg/L (1 Finnish MRSP, 0.25 mg/L; 1 U.S.A. MSSP, 2 mg/L; 1 Finnish MRSP and 1 U.S.A. MSSP, 4 mg/L; Table 2). The MICs for fusidic acid and chlorhexidine did not differ between MRSP and MSSP within groups of isolates from each country (fusidic acid U.S.A., Germany, SEUK *P* = 1.000, Finland *P* = 0.408; chlorhexidine U.S.A, Finland, SEUK *P =* 1.000, Germany *P =* 0.152) except in the NUK (*P* < 0.005 for both fusidic acid and chlorhexidine).

Twenty-seven of all 578 staphylococci, had fusidic acid MIC ≥64 mg/L and thus underwent *fusA* and *fusE* sequencing (5 MRSA SEUK, 4 MSSP NUK, 4 MRSP NUK, 1 MRSP SEUK, 1 Finnish MRSP, 12 Finnish FA-R MRSP). All eight internal primers designed to sequence the 2100 bp product of *fusA* aligned to *S. pseudintermedius*. Although two primers (FusA_Int_D_F and FusA_Int_F_R; Table 3) did not align with *S. aureus* isolates, the whole gene sequence was obtained using the remaining six primers in 3 / 5 MRSA isolates. In the remaining two MRSA isolates, mutation analyses were prevented by failure to amplify *fusA*.

**Table 3:** Six custom primers designed and used for coverage of entire *fusA* PCR amplicon of staphylococci for Sanger sequencing, alongside previously described forward and reverse primers (reference [34])

Primer Name	Primer Sequence	Forward / Reverse	Base pair sequenced from
FusA_Int_A_F	CGCCAACTCACGTGAAGAAA	Forward	1077
FusA_Int_B_R	ATTGACCACGACCACCAGAT	Reverse	1516
FusA_Int_C_R	TGCTTCACGTGCTTCTTCAG	Reverse	639
FusA_Int_D_F	CCAATCGGTGCTGAAGATGA	Forward	493
FusA_Int_E_F	ATCTGGTGGTCGTGGTCAAT	Forward	1497
FusA_Int_F_R	TGAGTTGGCTGTCATTTGTA	Reverse	1086
FusA_F^a^	TTTACCCTGAGTGTGTTCT	Forward	94
FusA_R^a^	TACATTTAAGCTCACCTTGT	Reverse	2256

^a^Previously described primers (reference [34])

Of the remaining 25 isolates with an MIC ≥64 mg/L (3 MRSA, 4 MSSP NUK, 4 MRSP NUK, 1 MRSP SEUK, 1 Finnish MRSP, 12 Finnish FA-R MRSP), 24 had at least one *fusA* mutation (Table 4); one MRSA isolate had none*.* All mutations observed represented non-conservative substitutions (Table 4). No *fusE* mutations were detected in any of the tested isolates (fusidic acid MIC ≥64 mg/L).

**Table 4:** Mutation sites detected in *fusA* in two methicillin-resistant *Staphylococcus aureus* (MRSA), 18 methicillin-resistant *S. pseudintermedius* (MRSP) and 4 methicillin-sensitive *S. pseudintermedius* (MSSP) isolates

Amino acid substitution	Nucleotide substitution	No. of isolates	Fusidic acid MIC (mg/L)
L461K	TTA - > AAA	2 (SEUK MRSA)	384^a^
I461K	ATT - > AAA	1 (NUK MSSP)	> 64
V90I / A376V / I461T	GTA - > ATA / GCA - > GTA / ATT - > ACT	1 (NUK MSSP)	> 64
		20 (*n* = 12 FA-R Finland MRSP; *n* = 1 Finland MRSP, *n* = 2 NUK MSSP, *n* = 4 NUK MRSP, n = 1 SEUK MRSP)	64

*MIC* minimum inhibitory concentration, *SEUK* South-East U.K, *NUK* North U.K.

^a^MIC determined as part of previous study (reference [52])

Of the plasmid-mediated fusidic acid resistance genes, *fusB* and *fusC* were detected in the collection but *fusD* was not (Table 1). In *S. pseudintermedius, fusB* was detected in 2 isolates (5%) of the Finnish FA-R MRSP; *fusB* was not detected in isolates from any other region, nor in *S. aureus.* In Finnish *S. pseudintermedius, fusC* was quite regularly detected (13/40 FA-R MRSP, 9/49 MRSP, 5/50 MSSP), as well as being found in 7/49 NUK MRSP. It was not detected in *S. pseudintermedius* from any other region. In *S. aureus, fusC* was detected in 2/50 SEUK MSSA.

Both *fusB* carrying isolates had MICs of 8 mg/L (Table 1). In 32 isolates (89%) carrying *fusC*, the fusidic acid MIC was 4–16 mg/L (Table 1). However, the other four isolates carrying *fusC* (1 NUK MRSP, 2 Finnish MRSP, 1 Finnish MSSP) had a fusidic acid-sensitive phenotype (MIC = 0.06 mg/L) (Table 1).

The only MRSA isolate with a fusidic acid MIC of 64 mg/L that had no mutation in either *fusA* or *fusE*, did not carry *fusB*, *fusC* or *fusD* either. Similarly, 51 isolates (1 MSSA, 34 MRSP, 16 MSSP) with ‘low-level’ fusidic acid resistance (MIC 4–16 mg/L) [28] did not carry *fusB, fusC* or *fusD.*

The chlorhexidine resistance determinants *qacA/B* and *smr* were not detected in any isolates from Germany or Finland, nor in *S. pseudintermedius* from SEUK (Table 2). In *S. pseudintermedius* from the U.S.A., 3/50 MRSP isolates and 1/51 MSSP isolates carried the *smr* gene; 1 NUK MSSP isolate (out of 50) carried *qacA/B.* In *S. aureus* (all SEUK), 1 MSSA carried *qacA/B* and 1 carried *smr*. Presence of *smr* related to chlorhexidine MICs of 0.5–4 mg/L and presence of *qacA/B* related to chlorhexidine MICs of 2–4 mg/L (Table 2).

No single isolate carried more than one of the resistance determinants investigated.

## Discussion

The same acquired resistance genes (*fusA* mutations, *fusB, fusC, qacA/B* and *smr*) that have been previously described in human-derived *S. aureus* [32–34] were found in canine-derived *S. pseudintermedius* and *S. aureus* in this study. However, for some of these genes, evidence of their association with increased fusidic acid and chlorhexidine MICs remains inconclusive.

For *S. aureus*, chromosomal mutations in *fusA* have been shown experimentally to elevate the MIC of fusidic acid by up to 32-fold [30], causing ‘high-level’ fusidic acid resistance in clinical isolates. The results from this study now support a previous report on a single isolate [35] that this is also the case for *S. pseudintermedius,* as *fusA* mutations were detected in isolates from the SEUK (MRSP), NUK (MRSP and MSSP) and Finland (MRSP). Whether *fusA* mutations play a role in ‘low-level’ resistance (MIC 4–16 mg/L) [28] remains to be investigated, particularly since there were 51 isolates with MICs compatible with ‘low-level’ resistance that did not carry *fusB-D* [28]. Failure to amplify *fusA* in two MRSA isolates might reflect mutation(s) at primer-binding sites; this could be evaluated by whole genome sequencing.

The single canine-derived MRSP previously reported with *fusA* mutations [35], showed substitutions at the same three sites (V90I, A376V and I461V) as those found in 20 of the 24 isolates with *fusA* mutations in this study. A novel substitution at one of these sites (I461T), likely related to reduced fusidic acid susceptibility, was shown in MRSP from Finland, NUK and SEUK. The other two amino acid substitutions found within *fusA* during this study (V90I, A376V) were at positions that are conserved between fusidic acid-susceptible *S. aureus* and *S. pseudintermedius*, and mutations at these sites have been previously described in European *S. aureus* [27, 30, 32, 35]. Substitution at position 90 (V - > I) has previously been shown to be unrelated to fusidic acid resistance in *S. pseudintermedius* when found on its own [35], and could have a compensatory effect to counteract fitness cost associated with other mutations [50]. The novel identification of the same mutations in three MSSP isolates from the NUK could be due to loss of methicillin resistance. This has been previously demonstrated in *S. aureus,* due to fitness costs of carrying some SCC*mec* cassettes [51, 52], and is more likely than an identical set of three single nucleotide polymorphisms arising in a separate lineage. Two canine-derived MRSA in this study had a single mutation (L461K), which has been previously described in human-derived *S. aureus* [32, 34]*,* reflecting that canine-derived MRSA isolates usually represent transfer of successful human-hospital-associated lineages [53] into the canine population.

This is the first description of *fusB* in *S. pseudintermedius* resulting in ‘low-level’ fusidic acid resistance, in parallel to that previously described in *S. aureus* [28]*.* The presence of *fusB* in a new staphylococcal species suggests that there may have been genetic transfer of plasmids between staphylococci. Previous studies indicate that this may be a rare occurrence due to the difference in restriction modification systems amongst staphylococci [35, 54]. However, the potential for further genetic transfer of resistance determinants between *S. aureus* and *S. pseudintermedius,* and amongst *S. pseudintermedius* lineages*,* as previously shown for *mecA* amongst staphylococci [44–47]*,* highlights a risk to human health from any increase in resistance in veterinary-derived staphylococci and vice-versa. The presence of the same plasmid-mediated resistance in different species could also be evidence of a common ancestor for these genes (such as *fusB* and *fusC* which show protein homology), as has been previously described for the SCC*mec* of staphylococci [46].

Whilst in the majority of cases the presence of *fusC* was related to ‘low-level’ fusidic acid resistance, as described in *S. aureus* carrying *fusC* [28], we report, for the first time, four *S. pseudintermedius* isolates with a susceptible phenotype (MIC = 0.06 mg/L) despite presence of *fusC*. This may reflect the fact that in previous studies, the presence of this gene has been investigated only in phenotypically fusidic acid-resistant (fusidic acid MIC ≥1 mg/L) isolates [32–34]. Phenotypic susceptibility in the presence of *fusC* could be due to a chromosomal rather than a plasmid location of *fusC* [55]*,* low copy number of *fusC-*containing plasmids, or due to non-expression of the gene. It may also question the relevance of *fusC* in reducing susceptibility to fusidic acid.

The low fusidic acid MICs in canine-derived *S. pseudintermedius* from the U.S.A. corresponds to its lack of use in veterinary medicine, whereas MICs were higher in isolates from the U.K and Finland which have had licensed fusidic acid available to veterinarians for a number of years. This mirrors the MICs of human-derived staphylococci which tend to reflect fusidic acid use [32, 33]. Although MIC_50_ remained low in most regions tested (with the exception of NUK MRSP), in our study the identification of genetic resistance determinants correlated with raised MIC_90_ in isolates from Finland and the U.K., matching the apparently bimodal distribution of MICs above and below a ‘resistance’ cut-off. The prevalence of *fusB* and *fusC* in the European canine-derived staphylococci in this study is broadly comparable to that seen in human-derived *S. aureus* [32], with variation in gene presence between isolates originating from differing European countries.

This study represents the first description of *qacA/B* and corroborates previous reports of *smr* in *S. pseudintermedius* [36]*,* and supports recent reports of *qacA/B* in canine-derived *S. aureus* isolates*,* although at lower frequencies than previously identified in human-derived *S. aureus* (6% in U.S.A. MRSP in this study, compared to 8.3–63% previously described) [56–59]. However, neither *qacA/B* nor *smr* appeared related to high chlorhexidine MICs, similar to what has been described for human-derived *S. aureus* [38, 41] and *S. epidermidis* [60], and in a limited collection of *S. pseudintermedius* [36]*.* This raises the possibility of different, and as of yet unknown, mechanisms being responsible for raised chlorhexidine MICs. Chlorhexidine MICs remained remarkably uniform within the same geographical region, represented by MIC_50_/MIC_90_ within one dilution of each other. Although statistically significant, the difference in the MIC between some geographical regions was no more than one to two dilutions, which is unlikely to represent a clinically significant variation in efficacy of chlorhexidine-based products. The MIC range of chlorhexidine across all geographical regions tested correlated closely with that previously described for *S. aureus* [37, 38], potentially reflecting relatively uniform use of chlorhexidine-based products globally in both human and veterinary medicine.

The increasing interest in the use of topical therapy for canine pyoderma amidst efforts towards good antimicrobial stewardship has highlighted the absence of clinically relevant breakpoints for topically applied agents, such as fusidic acid and chlorhexidine. The concentrations of fusidic acid obtained within the skin 24 h after application to a canine skin model (of the order of 2000 mg/L) [61], are approximately 1000-fold higher than the EUCAST breakpoint for fusidic acid for staphylococci (derived for systemic administration in humans) [62]. In each of the geographical regions studied, topical fusidic acid therapy would be expected to achieve concentrations in canine skin far exceeding the majority of MICs described in this study [61], despite presence of resistance determinants in the staphylococcal population tested. These observations highlight the questionable relevance of routine susceptibility testing with current conventional protocols when assessing topical treatment options. There is an urgent need for development of breakpoints that might usefully predict antimicrobial efficacy of topically applied drugs in surface and superficial skin infections [63], to prevent the unwary clinician being misled by laboratory application of existing breakpoints, developed for systemic therapy, that leads to reports of ‘resistance’.

## Conclusions

Resistance determinants associated with tolerance to fusidic acid (*fusA* mutations, *fusB* and *fusC*) were detected at a low rate in canine-derived staphylococci in this study. Conversely, the presence of *qacA/B* and *smr* appeared to have no effect on chlorhexidine MIC. The low fusidic acid MICs and the lack of fusidic acid resistance determinants in isolates from the U.S.A. compared to the other geographical regions was not surprising, given that fusidic acid is not authorised for use in dogs in the U.S.A. In addition, the overall low prevalence of resistance genes in this collection of 578 mostly clinical staphylococcal isolates, and the corresponding low MICs for fusidic acid and chlorhexidine, indicate good continued antibacterial efficacy of these agents. Further clinical studies to provide good evidence for *in vivo* efficacy of these antimicrobials in canine surface and superficial pyoderma should be encouraged. Further investigations are now needed to elucidate further the role of *fusC, qacA/B* and *smr,* and to investigate for novel resistance characteristics that may be of relevance to facilitate future resistance monitoring and to guide appropriate use of these valuable agents.

## Methods

### Bacterial isolates

A total of 578 coagulase-positive staphylococci (100 *S. aureus* obtained in 2005–07 and 478 *S. pseudintermedius* obtained in 2010–16) isolated from dogs were included from four countries: U.K. (split into South-East [SEUK] and North [NUK] as previously defined) [48], Germany, Finland and the U.S.A. (Table 5).

**Table 5:** Geographical origin of canine-derived staphylococci used in this study

Geographical location	South-East U.K.^a^	North U.K.^b^	Germany^c^	Finland^d^	U.S.A.^e^	*Total*
MRSP	47	49	24	49	50	*219*
FA-R MRSP	0	0	0	40	0	*40*
MSSP	44^f^	50	24	50	51	*219*
MRSA	50	0	0	0	0	*50*
MSSA	50	0	0	0	0	*50*
*Total*	*191*	*99*	*48*	*139*	*101*	*578*

*MRSP* methicillin-resistant *S. pseudintermedius, FA-R* fusidic acid resistant as defined by disk diffusion testing, *MSSP* methicillin-sensitive *S. pseudintermedius, MRSA* methicillin-resistant *S. aureus, MSSA* methicillin-sensitive *S. aureus*

All of clinical origin except ^f^ which are of clinical (*n* = 3) and carriage (*n* = 41) origin

From the authors’ collections: ^a^SMF, RB, AL; ^b^DT; VMS; ^c^AL; ^d^MR, TG; ^e^SCR, KO

All isolates were collected from canine infections (with the exception of SEUK MSSP, which were both clinical and carriage isolates) (Table 5). In order to investigate isolates with a wide range of fusidic acid susceptibility, an extra 40 clinical MRSP from Finland were included (Finnish FA-R MRSP), which had been determined as fusidic acid-resistant by disk diffusion testing (by MR and TG) interpreted using the Finnish FiRe criteria (Table 5) [64, 65]. Species identification and methicillin resistance were confirmed (by SMF and AL) by both phenotypic [66] and genotypic methods (for species-specific *nuc* and methicillin resistance *mecA*) [67–69].

### MIC determination

Fusidic acid and chlorhexidine MICs were determined in duplicate using an agar dilution method (CLSI VET01-A4) [70]. Fusidic acid and chlorhexidine MICs for isolates from SEUK and Germany, and chlorhexidine MICS for NUK isolates, have been reported previously [48, 49]. Stock solutions of fusidic acid sodium salt (F0881, Sigma-Aldrich Inc., Gillingham, U.K.) and chlorhexidine (C9394, Sigma-Aldrich Inc.) at 10 x final concentration, adjusted for drug potency, were prepared in distilled water [70]. Final concentrations of the active fraction ranged from 0.015–64 mg/L for fusidic acid and 0.125–64 mg/L for chlorhexidine, based on previous experience [48, 49]. Discrepancy between the duplicate MICs was accepted, provided they varied by only one dilution; in these cases, the higher value was recorded as the final MIC as a conservative measure. For quality control purposes *S. aureus* subsp. *aureus* (ATCC® 25923™), *S. pseudintermedius* (LMG 22219) and *S. intermedius* (ATCC® 29663™) were included. Isolates were defined as resistant or susceptible to fusidic acid using EUCAST breakpoints [21]; chlorhexidine breakpoints remain unreported.

### Identification of resistance genes and mutations

Extraction of DNA was performed using a commercial purification kit (Bacterial Genomic DNA Purification kit, Edge BioSystems, Gaithersburg, MD, U.S.A). Each isolate’s DNA was screened by PCR for the presence of *fusC* [34]*, fusD* [34]*, qacA/B* [71] and *smr* [72] using primers and methods as previously described. To detect *fusB*, a previously described PCR reaction [55] was optimised by the addition of 1.5 mM MgCl_2_ to the PCR mastermix (total 16.5 mM MgCl_2_).

In isolates with fusidic acid MIC ≥64 mg/L, *fusA* and *fusE* were amplified by PCR and then sequenced using the Sanger method (Source BioScience, Nottingham, U.K.) to identify mutations. A previously described method was used for PCR amplification of *fusE* [33]*.* The PCR for amplification of *fusA* [34] was optimised by increasing the elongation time from 2 to 3 min. Four forward and four reverse primers, comprising six custom designed using the *S. pseudintermedius* ED99 genome sequence [35], and two previously described primers [34] were used for sequencing of the complete gene (Table 3)*.* Nucleotide and translated amino acid sequences were aligned to control *S. aureus* (ATCC® 29663™) and *S. (pseud)intermedius* (ATCC® 25923™, LMG 22219) *fusA / fusE* sequences using EMDL-EBI Clustal Omega Multiple Sequence Alignment Tool [73] and BLAST analyses (https://blast.ncbi.nlm.nih.gov/Blast.cgi).

Positive controls for each PCR comprised DNA extracts from strains carrying the desired genes. These were *fusA/fusE, S. aureus* subsp. *aureus* [ATCC® 25923™] and *S. intermedius* [ATCC® 29663™]; *fusB, S. aureus* B30 [74]; *fusC, S. aureus* MSSA 476 [75]; *fusD, S. saprophyticus* subsp. *saprophyticus* [ATCC® 15305™]; *qacA/B, S. aureus* Mu50 [76]; *smr, S. aureus* E37 [74]).

### Statistical analysis

The MICs (dependent variable) for fusidic acid and chlorhexidine were compared between MRSP and MSSP (independent variables) within each geographical region using the Kruskal-Wallis test. Since MRSP and MSSP MICs did not vary within any of the regions, *S. pseudintermedius* MICs (dependent variable) were subsequently compared between regions (independent variable) using the Kruskal-Wallis tests with post hoc comparisons using Mann-Whitney U-tests with Holm-Bonferroni adjustments. These statistical analyses were performed using SPSS version 21 (IBM UK Ltd., Portsmouth, U.K.), with *P* < 0.05 denoting significance.

## Abbreviations

CHX: Chlorhexidine
FA: Fusidic acid
MIC: Minimum inhibitory concentration
MR: Methicillin-resistant
MS: Methicillin-susceptible
NUK: North U.K.
PCR: Polymerase chain reaction
PFGE: pulsed-field gel electrophoresis
SA: *Staphylococcus aureus*
SEUK: South-East U.K.
SP: *Staphylococcus pseudintermedius*
